# Role of Clinical Risk Factors and B-Type Natriuretic Peptide in Assessing the Risk of Asymptomatic Cardiotoxicity in Breast Cancer Patients in Kazakhstan †

**DOI:** 10.3390/diagnostics13233557

**Published:** 2023-11-28

**Authors:** Zhenisgul Tlegenova, Saule Balmagambetova, Bekbolat Zholdin, Gulnara Kurmanalina, Iliada Talipova, Arip Koyshybaev, Gulmira Sultanbekova, Mira Baspayeva, Saule Madinova, Kulparshan Kubenova, Aiganym Amanova, Amin Tamadon

**Affiliations:** 1 Department of Internal Diseases-2, West Kazakhstan Marat Ospanov Medical University, Aktobe 030019, Kazakhstan; zhenisgul.tlegenova@zkmu.kz (Z.T.); b.zholdin@zkmu.kz (B.Z.); kurmanalina.g@zkmu.kz (G.K.); talipova.i@zkmu.kz (I.T.); g.sultanbekova@zkmu.kz (G.S.); amanova.a@zkmu.kz (A.A.); 2 Department of Oncology, West Kazakhstan Marat Ospanov Medical University, Aktobe 030019, Kazakhstan; arip.koyshybaev@zkmu.kz; 3 Chemotherapy Division at University Medical Center, West Kazakhstan Marat Ospanov Medical University, Aktobe 030019, Kazakhstan; mira.baspayeva@zkmu.kz (M.B.); saule.madinova@zkmu.kz (S.M.); 4 Clinical Laboratory at University Medical Center, West Kazakhstan Marat Ospanov Medical University, Aktobe 030017, Kazakhstan; kkubenova@mail.ru; 5 Percia Vista R&D Co., Shiraz, Iran; amintamaddon@yahoo.com; 6 Department for Scientific Work, West Kazakhstan Marat Ospanov Medical University, Aktobe 030019, Kazakhstan

**Keywords:** cardiotoxicity, chemotherapy, risk factors, biomarkers, B-type natriuretic peptide, predictive models, breast cancer, Kazakhstan

## Abstract

The asymptomatic progression of chemotherapy-induced cardiotoxicity poses a significant risk to breast cancer patients. In the present single-center cohort study, a predictive model for evaluating the risk of cardiotoxicity during or by the end of chemotherapy was designed. The risk-prediction nomogram was delineated and assessed. In total, 34 patients out of 120 developed asymptomatic cardiotoxicity (28.3%). Of six explored biomarkers, only B-type natriuretic peptide showed a reliable pattern of incremental increase, revealing statistical significance between cardiotoxicity “+” and “−” groups by visit 4 or by the 9th month of monitoring (*p* 0.006). The following predictors were included in the model: age, hypertension, diabetes mellitus, baseline glomerular filtration rate, 6 min walk test measured at visit 4, BNP values at visit 4, left ventricular ejection fraction levels at visit 4, a total dose of radiotherapy received, and anthracycline cumulative doses. The model’s AUC was 0.72 (95% CI 0.59; 0.86), evidencing the satisfactory predictive ability of the model; sensitivity 100% (95% CI 90.36; 100.0) at a specificity of 66.67% (95% CI 50.33; 79.79); PPV 54.1% [95% CI 47.13; 60.91]; PVN 100% [95% CI 94.64; 100.00]. The calibration plot showed satisfactory agreement between predicted and actual chances (*p* = 0.98). The designed model can be applied in settings lacking speckle tracking echocardiography.

## 1. Introduction

Breast cancer (BC, all abbreviations are explained in the Abbreviations section) accounts for 12.5% of all new annual cancer cases worldwide, making it the most common cancer globally [1]. By 2040, the burden of breast cancer is predicted to increase to over 3 million new cases and 1 million deaths yearly [2]. In Kazakhstan, breast cancer is also the leading cause of cancer morbidity and mortality among women. The all-cause mortality rate for BC within 2014–2019 was 16%. The prevalence rate increased to 50.6 in 2019, and the incidence rate varied from 4.5 per 10,000 population to 7.3 [3].

Nonetheless, the number of cancer survivors continues to grow in the United States and other countries, primarily due to improved strategies for early detection and advances in antitumor pharmacological treatment, particularly chemotherapy. However, most cancer survivors must cope with the physical effects of cancer and its treatment, leading to functional and cognitive impairments [4,5]. It has been established that BC survivors are three times more likely to develop heart failure (HF) within five years of cancer diagnosis than the general population [6]. According to Chinese researchers, the most vulnerable period is 30–64 years, and the risk peaks within the first months after diagnosis [7]. This worrisome trend is mainly explained by the cardiotoxic effects of chemo- and radiotherapy. Cardiovascular complications in cancer patients include coronary artery disease, hypertension, QT prolongation, arrhythmias, stroke, peripheral arterial disease, valvular heart disease, pericardial disease, and venous thromboembolism. Among the cardiotoxic effects of chemotherapy, now defined in the literature as cancer therapy-related cardiac dysfunction (CTRCD), the development of left ventricular dysfunction (LVD) is the most common and severe [8].

Increasing insight into CTRCD pathophysiology induced by anticancer therapy has allowed its classification by highlighting the two groups. CTRCD type 1 is characterized by irreversible myocardial damage, while CTRCD type 2 is characterized by reversible myocardial dysfunction. Anthracyclines, the most widely used class of chemotherapy agents in BC treatment, induce cardiotoxicity type 1, which is dose-dependent [9]. The incidence of HF is estimated to reach 5% at a cumulative dose of 400 mg/m^2^ of anthracyclines, increasing to 16% at 500 mg/m^2^, 26% at 550 mg/m^2^, and 48% at 700 mg/m^2^ [10]. Trastuzumab, a targeted therapy in HER2-positive BC, leads to CTRCD type 2 [11]. Additionally, radiotherapy, often applied to the chest area during BC treatment, can harm the heart and its surrounding structures, increasing the risk of HF development. Compared to other forms of cardiomyopathy, such as ischemic or dilated cardiomyopathy, the prognosis is poor, with a 5-year survival rate of <50% [12]. To address this growing problem, the medical community has turned its attention to the importance of early detection and intervention in cases of chemotherapy-induced cardiotoxicity. It has been shown that in the early stages of CTRCD, prompt initiation of treatment with angiotensin-converting enzyme (ACE) inhibitors and beta-blockers potentially prevents further deterioration of cardiac function [13,14]. In addition to known cardiac protectors, various co-adjuvant medications have been introduced in oncologic practice to mitigate the antineoplastic and accompanying disease treatment consequences, such as high-intensity statins, antidiabetics, and anthelmintics [15,16].

CTRCD asymptomatic progression leads to a significant risk to BC patients. There is a need for careful monitoring of heart function using reliable and cost-effective tools to detect asymptomatic left ventricular myocardial dysfunction. The 2022 Cardio-Oncology Guidelines contain an updated definition of asymptomatic CTRCD, classifying it as mild (left ventricular ejection fraction (LVEF) ≥ 50% and new relative global longitudinal strain (GLS) reduction >15% compared to baseline and/or new elevation of cardiac biomarkers); moderate (≥10% to 40–49% reduction in LV ejection fraction or <10% to 40–49% reduction in LV ejection fraction and relative GLS reduction >15% or elevated cardiac biomarkers); or severe (LV ejection fraction <40%), aligning with the European Society of Cardiology (ESC) HF guiding principles classification [17]. These guidelines also comprehensively summarize the role of clinical risk factors in assessing the potential cardiotoxicity of upcoming chemotherapy and highlight the strategy for stratifying patients’ cardiovascular risk at baseline.

Many researchers are currently focused on applying cardiac markers as they are simple and relatively cost-effective compared to imaging methods, particularly speckle tracking echocardiography (STE) or GLS assessment. In addition, including biomarkers in the algorithms of CTRCD detection allows for a comprehensive evaluation of cardiac function. Studies have shown that elevated levels of the primary cardiac biomarkers, troponin and B-type natriuretic peptide, are significantly associated with HF and asymptomatic LVD within cancer treatment [18,19,20,21,22,23]. Several new potential biomarkers, such as C-reactive protein (CRP), soluble suppression of tumorigenicity 2 (sST2), galectin-3 (Gal-3), myeloperoxidase (MPO), placental growth factor (PIGF), tumor growth differentiation factor (GDF-15), and microRNAs, hold promise for predicting cardiovascular disease not only in cancer patients but in cases of other chronic diseases [24,25]. Some of these biomarkers (CRP, Gal-3, MPO) reflect such aspects of heart pathophysiology as oxidative stress, inflammation, and fibrosis [26]. High values of CRP can predict mortality in patients with acute decompensated heart failure one year after discharge [27]. CRP has recently been shown to have significant predictive value in tumor immunotherapy as a prognostic biomarker for immune checkpoint inhibitors (ICIs) treatment [28,29]. Gal-3 is involved in the occurrence and development of cardiac fibrosis, HF, and atherosclerosis [30]. MPO is known to be an independent predictive factor of 1-year mortality in acute HF patients [31]. Despite many papers demonstrating the high efficacy of biomarkers in CTRCD diagnosis, some researchers have not confirmed this point of view. Reportedly, the longitudinal LVEF trajectory, not highly sensitive primary cardiac markers, allows for a dynamic assessment of cardiotoxicity risk in early BC [32]. The lack of a unified position among researchers on the issue of the best method for diagnosing CTRCD conditions implies the involvement of all obtained data—clinical risk factors, imaging methods, and biomarkers with high performance.

The present study investigated the ability to predict asymptomatic CTRCD by assessing clinical factors and conventional biomarker panel performance. Our efforts were directed toward designing a predictive model to evaluate the risk of potential cardiotoxicity during or by the end of treatment to provide timely administration of cardioprotective medications and chemotherapy modification if needed. Predictive models significantly contribute to developing diagnostic and treatment algorithms, assisting in balancing the need to treat cancer and maintain heart health.

## 2. Materials and Methods

### 2.1. Study Design and Ethical Approval

This observational single-center study consisted of two parts: retrospective and prospective. All available information collected in database research (retrospective part—data from the Aktobe Regional Oncologic Registry as of 2018–2019) was published in 2022 [33]. The prospective cohort stage started in September 2021. The study was approved by the Bioethics Committee of the West Kazakhstan Marat Ospanov Medical University (Ref. No. 7, 9 September 2020) and posed no risk for participating individuals due to its observational nature. The patients’ rights were protected. Participation in the study was voluntary. Women were examined after explaining the research purposes and procedures and informed consent signing.

### 2.2. Patient Selection

**Figure 1:**
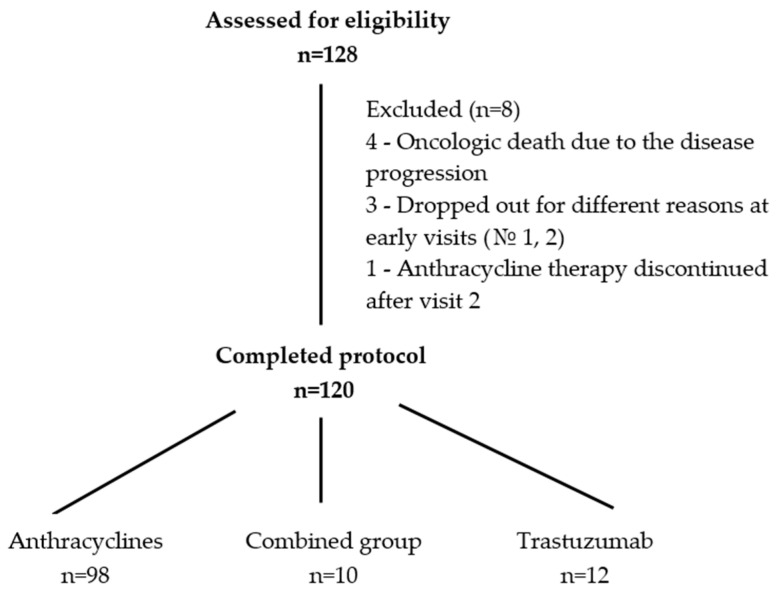
Flowchart of patient selection.

Between September 2021 and August 2022, we consecutively recruited a prospective cohort of eligible women newly diagnosed with BC on admission to the chemotherapy division of the University’s Medical Center for treatment with anthracyclines and/or trastuzumab. The overall sample was *n* 128. All women after surgery were provided with the immunohistochemistry (IHC) analysis. Inclusion criteria were: verified diagnosis of C50 at any stage (if eligible for neoadjuvant or adjuvant chemotherapy, not palliative); any age (18+); targeted therapy upon the confirmed HER2-positive status, and/or anthracyclines administration; and Simpson LVEF > 40% without symptoms of heart failure established within 30 days before admission (as known, LVEF < 40% is a contraindication for using anthracyclines and trastuzumab). Patients who were administered targeted therapy with kinase inhibitors (KIs) or ICIs were excluded. We also included patients who received radiation treatment before chemotherapy, as many schemes implied combined treatment. Exclusion criteria: patients showing LVEF < 40% by Simpson or having established cardiac dysfunction (congestive heart failure or coronary heart disease progression) or severe comorbidities. The overall number of eligible participants was 128, but the eventual sample consisted of 120 individuals (see Figure 1).

### 2.3. Types of Collected Data and Visit Intervals

The research implied five visits, including an initial appointment and then every three months. Data to be collected included general information (demographic, medical history, menopause, BMI, heredity, presence of lifestyle risk factors); tumor features (BC clinical stage and types, tumor histotype, immunohistochemistry (IHC) data); clinical data (Charlson comorbidity index—disease and scores, presence/absence of arterial hypertension, diabetes mellitus, etc.); previous treatment (type of surgery, breast radiotherapy—radiation side, total focal dose received); the administered chemotherapy (pharmaceuticals, dose, and regimen); and cardiac protectors (if prescribed). During the visits, the following data were collected: systolic/diastolic blood pressure (SBP/DBP), 6 WT (6 min walk test, meters), selected clinical and biochemical lab tests (according to the “Breast Cancer” in-country protocol dated 1 March 2019, No. 56), echocardiographic data (values of left ventricular ejection fraction (LVEF) and speckle tracking data), and biomarkers assays. The values of six conventional biomarkers: cardiac troponin (cTnI), B-type natriuretic peptide (BNP), C-reactive protein (CRP), myeloperoxidase (MPO), galectin-3 (Gal-3), and D-dimer were explored, as stated in the study protocol [34]. All these data were recorded on the patient’s registration card (IRC, Appendix No. 5 to the protocol). The patient’s IRC example and other Supplementary Materials are in a publicly available repository, osf.io [http://osf.io/nykmw/ (accessed on 10 June 2022)]. In addition, the following data were subject to be recorded in IRC: presence/absence of CTRCD, if yes—type, form, time of emergence; any other complications during chemotherapy courses; chemotherapy outcomes, including modifications due to CTRCD; interruption due to CTRCD; changes due to non-CTRCD complications; interruption due to non-CTRCD complications; completeness of chemotherapy (%); and death (cause; date).

### 2.4. Allocation of Patients by Risk Groups

According to the 2022 ESC (European Society of Cardiology) Guidelines on cardio-oncology, the stratification of cancer patients by cardiovascular risk groups is one of the crucial steps in their management [17]. During baseline clinical examination (visit 1), the potential risk of CTRCD emergence was calculated. In line with the current strategy, the risk scores were calculated considering all possible risk factors—existing (recorded) cardiovascular diseases, the toxicity of chemotherapy prescribed, and lifestyle risk factors. If there was one moderate risk factor or an absence of risks at the moment of examination, the patient was allocated to the low-risk group. If two to four scores of moderate risk were present, they were assigned to the moderate risk group. If more than five scores of moderate risk or at least one high-risk factor were revealed, they were allocated to the high-risk group. Respectively, patients with one very-high-risk factor were allocated to the very-high-risk group. “Very high” risk means the presence of existing chronic heart failure or dilated cardiomyopathy; “high” risk includes previous severe valvular heart disease, past myocardial infarction and/or revascularization, baseline LVEF < 50%, stable angina, or preceding radiation therapy. Thus, all 128 patients were allocated into risk groups. Patients with very high, high, and moderate risk were given medications according to indications: ACE inhibitors/ARBs (angiotensin II receptor blockers), beta-blockers, statins, trimetazidine/analogs, and others. As stated in the protocol, we strived to gain a satisfactory level of adherence in those with prescribed cardioprotectants [34]. For all enrolled participants, a particular individual form, “Cardiovascular risk stratification for upcoming chemotherapy treatment,” was filled out (Appendix No. 6 placed in the repository, [http://osf.io/nykmw/ (accessed on 10 June 2022)]). The patient’s risk scores were stated in this form based on the defined hazard of risks and individual risk levels.

### 2.5. Chemotherapy Treatment

As stated above, we enrolled only the patients who started on trastuzumab and/or anthracyclines. The chemotherapeutic regimen was selected according to the disease status and risk factors. Most of the patients who were administered anthracyclines received AC or AC-T regimens. The AC regimen was 60 mg/m^2^ doxorubicin and 600 mg/m^2^ cyclophosphamide, used on the first day and administered every 21 days for four courses. The AC-T regimen was the same doses of AC during the first four courses, after which T (paclitaxel or docetaxel) was given at 90 mg/m^2^ for four courses (every 21 days). Trastuzumab (Herceptin) was presented at standard regimen every three weeks for up to 18 courses, with an initial dose of 8 mg/kg and then 6 mg/kg. In addition, a group of patients were administered a mixed regimen of trastuzumab and anthracyclines (HER2-positive cases). As known, administering adjuvant trastuzumab in a week cycle concurrently with an anthracycline-taxane chemotherapy regimen appears to be a preferable option to optimize its favorable effect in improving DFS (disease-free survival) and to prevent significantly higher risk for cardiotoxic effects [35]. The regimen was: an initial dose of 4 mg/kg as an intravenous infusion over 90 min, then at 2 mg/kg as an intravenous infusion over 30 min weekly during chemotherapy for the first 12 weeks (AC-TH regimen, paclitaxel or docetaxel) or 18 weeks (TCH regimen, docetaxel/carboplatin). Although we scheduled functional and laboratory measurements for 0, 3, 6, 9, and 12 months, we postponed the tests until the treatment intervals if patients received the treatment on these dates. Collection of biomarkers assays and functional and cardiac imaging examinations were performed only before the start of chemotherapy courses.

### 2.6. CTRCD Evaluation Criteria and Measurements

As we commenced research at the end of 2021, we applied criteria of cardiotoxicity taken from the position paper of 2020 to confirm the presence of CTRCD: a decrease in LVEF above 10% from baseline to a value of LVEF under 53% or a reduction in GLS deformation below 15% from the baseline value [36]. Accordingly, we measured and analyzed the following parameters:

1. The number of patients who developed cardiotoxic complications, including subclinical (asymptomatic) dysfunction, measured using LVEF monitoring (>10% decline from baseline to a value <53%) and GLS assessment (decrease >15% from baseline) after the chemotherapy onset through all groups;
2. Presence of increased values of the tests during chemotherapy treatment: cardiac troponin I (cTnI)—>0.3 ng/mL; brain natriuretic peptide (BNP)—>100 pg/mL; C-reactive protein (CRP)—>5 mg/L; myeloperoxidase (MPO)—>470 pmol/L; galectin-3 (Gal-3)—>28.7 ng/mL; and D-dimer—>0.5 mg/L (presented values were taken from the immunofluorescence analyzers’ manuals from the involved labs by request);
3. Time trends in the onset of increasing biomarker values;
4. The positive predictive value (PPV) and the negative predictive value (PVN) for all developed statistical models.

General blood and biochemical tests for this study were performed at the University Medical Center’s clinical lab. Some biomarkers’ detection (cTnI, BNP, CRP, D-dimer) was also performed in this lab using immunoassay analyzers and Finecare rapid quantitative tests (Guangzhou Wondfo Biotech Co., Guangzhou, China), in line with good laboratory practice (GLP). Galectin-3 and MPO assays were directed to a third-party collaborator, the “Olymp” labs network. The types of tests were the ELISA Galectin-3 S and ELiA MPO Well tests. For biomarker detection, all the blood samples were obtained at least 24 h before the start of therapy. The collected blood samples were placed in EDTA anticoagulation tubes, and the serum was obtained by centrifuging for 15 min at 4 °C at 1800× *g* to detect biomarker levels.

### 2.7. Cardiac Imaging

Two project staffers were responsible for transthoracic echocardiography (TTE) and speckle tracking. Cardiac imaging was performed using “automated function imaging (AFI)—automatic non-Doppler assessment of longitudinal strain of the left ventricular myocardium” software for VIVID E9 (EchoPAC version 2.4) and a M5-SD transducer, 1.5–4.5 MHz. Left ventricular end-diastolic and end-systolic volumes were calculated using Simpson’s approach to derive LVEF. The same specialists analyzed GLS according to currently accepted protocols [37,38]. The study area was corrected to cover the whole thickness of the myocardial wall. Measurements were performed from the apical 3-chamber (3C), 4-chamber (4C), and 2-chamber positions. When deriving apical positions, care was taken to ensure that the long axis of the ventricle was perpendicular to the plane of the mitral annulus in the LV apical views. Intra- and inter-observer coefficients of variation were not set for LVEF and GLS. CTRCD was defined as a >10% absolute decline in LVEF < 53% [36]. Subclinical myocardial LVD was considered to be a drop in GLS below (−) 18% in the range (0% to 17.9%) or a decrease in this indicator > 15% from baseline.

### 2.8. Statistical Analysis

Baseline characteristics were summarized in the present study using proportions for categorical variables (cancer treatment, etc.), and medians (interquartile range) were presented for continuous variables (biomarker values, etc.). The Pearson χ^2^ criterion was applied to identify intergroup differences for categorical variables. Quantitative variables were compared using the nonparametric U Mann–Whitney test regarding the dynamics in the level of biomarkers (0, 3, 6, 9, 12 months), etc., for two unrelated groups (with and without the development of cardiotoxicity). Univariate data analysis was used to directly investigate differences in the endpoint of interest (cardiotoxicity). To assess the influence of independent factors on the binary variable of response (cardiotoxicity), multiple logistic regression analysis (LRA) was used. To evaluate TTE and GLS performance toward detecting asymptomatic cardiotoxicity across CTRCD“+” and “−” groups, Friedman ANOVA by Rank test and Kendall concordance coefficient were applied.

The Gaussian family-based generalized linear model (GLM) was applied to characterize the biomarkers’ performance. Given the skewed distributions, all biomarkers were log2 transformed. Mean estimated changes from baseline were plotted over time to illustrate the temporal relationships between the biomarker levels and the cardiotoxicity groups (CTRCD“+”; CTRCD“−”). Mean changes were determined using repeated-measures linear regression estimated via generalized estimating equations. Each model was adjusted for the baseline values of the biomarker under consideration and the time since treatment initiation modeled nonparametrically using a cubic spline.

The risk-prediction nomogram was designed to predict asymptomatic cardiotoxicity. Seventy percent of patients were randomly selected as a training cohort to develop the predictive model, and 30% were allocated to an internal validation cohort. The discriminative power of the nomogram was assessed through three methods: means of the area under the receiver operating characteristic (ROC) curves (AUC), the Hosmer–Lemeshow goodness-of-fit test with a *p*-value of >0.05 indicating a good fit of data, and calibration plots between the model-predicted probabilities and the observed probabilities of cardiotoxicity [39,40]. Indices of sensitivity (Sn), specificity (Sp), positive predictive values (PPV), and negative predictive values (PVN) were calculated for a developed predictive model. PPV and PVN were computed by the commonly accepted methods [41,42]. The nomogram based on the univariate analysis, including clinically significant variables, was designed to evaluate the predictive abilities of selected biomarkers and clinical factors in detecting asymptomatic cardiotoxicity during or by the treatment end.

Two-sided levels < 0.05 were assumed to be statistically significant. For statistical processing, software packages SPSS (IBM, Armonk, NY, USA, v.25), Statistica (StatSoft, Inc., Tulsa, OK, USA, v. 10), and R 3.4.2 (R Foundation for Statistical Computing, Vienna, Austria) were used.

## 3. Results

### 3.1. Patient Characteristics

In total, 128 BC patients started on anthracyclines and/or trastuzumab from September 2022 to August 2023 were included in this study (Figure 1). Three participants dropped out of the study for different reasons at early terms (visits 1 or 2). Four others were excluded from statistical processing due to oncologic death without the signs of cardiotoxicity (the disease progression). Upon results of the first appointment, all patients were allocated into the cardiovascular risk groups by their individual scores. One woman, 58 years old, showed symptomatic CTRCD. The patient was referred to the very-high-risk group at baseline due to chronic heart failure. After enrollment in the study, she developed atrial fibrillation related to stable angina II. Before the start of chemotherapy, ACE inhibitor, beta-blocker, mineralocorticoid receptor antagonist (MRA), sodium-glucose cotransporter-2 (SGLT2) inhibitor, and oral anticoagulant were administered in line with the chronic heart failure (CHF) protocol. After three months of observation, LVEF fell from 51% to 41%, the patient’s condition deteriorated, and anthracycline therapy was canceled at a cumulative dose of 260 mg/m^2^. She was the only patient in the very-high-risk group due to CHF, and after anthracycline therapy discontinuation upon visit 2, this patient was excluded from the study. The remaining 120 patients completed all five visits. Of them, 12 received trastuzumab-based treatment, 10 received combined therapy, and 98—anthracyclines. According to baseline risk stratification, 67 (55.8%) patients were assigned to the low-risk group, 46 (38.3%) to the moderate-risk group, and 7 (5.8%) were referred to the high-risk group.

We analyzed the patients’ performances according to CTRCD definitions—echocardiography dynamics (LVEF, GLS), values of biomarkers under consideration (cTnI, BNP, CRP, D-dimer, Gal-3, and MPO), and clinical symptoms, including essential lab tests, through three treatment groups. We revealed 30 individuals with CTRCD in the anthracycline group (*n* 98; 30.6%) with cumulative anthracycline doses of 452.0 (350.0; 670.0) mg/m^2^; 3 individuals in the combined group (*n* 10; 30%) with the received doses of 540.0 (470.0; 550.0) mg/m^2^ anthracycline and 5420.0 (5320.0; 8840.0) mg of trastuzumab; and one patient in the trastuzumab group (*n* 12; 8.3%) with the received dose of 10158.0 mg. Overall, we detected 35 cases of CTRCD among 121 patients: 1 symptomatic (dropped out) and 34 subclinical. Asymptomatic cardiotoxicity was observed in 28.3% of the 120 patients under monitoring. The main characteristics of patients from the two groups, with developed cardiotoxicity (*n* = 34) and without (*n* = 86), are shown in Table 1.

Patients who eventually developed cardiotoxicity differed from those without signs of CTRCD by the presence of previously existing diseases that influenced the Charlson comorbidity index and patients’ cardiovascular risk scores measured at baseline. In addition, these individuals showed relatively worse baseline physical characteristics, such as a 6 min walk test and age. Considering the different mechanisms of CTRCD development in the groups of patients varying by type of treatment, we also compared patients by anticancer therapy parameters. No significant differences between the treatment groups regarding cardiotoxicity for 12 months of monitoring were found.

### 3.2. Evaluation of Imaging Methods’ Dynamics toward Asymptomatic Cardiotoxicity

To evaluate TTE/speckle tracking performance, we analyzed LVEF/GLS dynamics from visit to visit between CTRCD groups. Despite the absence of statistical difference at baseline between the CTRCD groups (GLS *p* 0.835; LVEF *p* 0.074, Table 1), these differences emerged further when monitoring from visit to visit. Data on GLS/LVEF dynamics are presented in Table 2.

Within the presented study, both imaging methods performed well, exhibiting significant distinctions between those who developed CTRCD (*n* 34) and those who did not (*n* 86) since visit 2, i.e., three months after the treatment outset. Nonetheless, although the differences were statistically significant for LVEF, they cannot be interpreted as clinically significant (as stated in the research protocol, only a decrease of 10% or less than 53% is considered essential to diagnose cardiotoxicity). Conversely, GLS since visits 3 and 4 (by the 6th–9th month of monitoring) demonstrated indices less than 15%, thus confirming the presence of CTRCD.

### 3.3. Dynamics of Biomarkers

**Figure 2:**
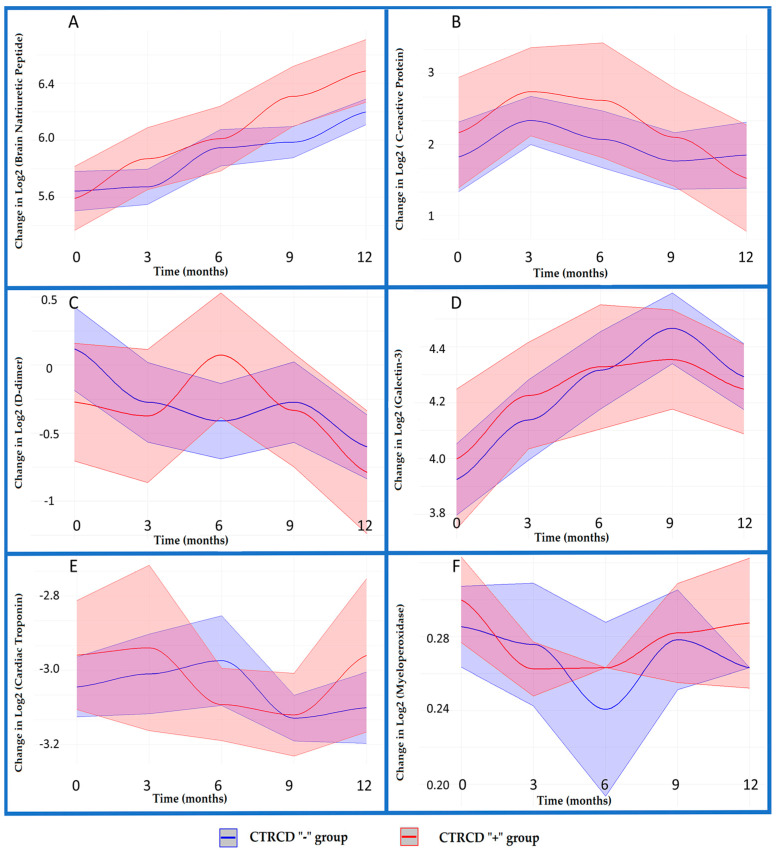
Biomarkers dynamics across CTRCD groups. (**A**) BNP; (**B**) CRP; (**C**) D-dimer; (**D**) Gal-3; (**E**) cTnI; (**F**) MPO (95% CI intervals for biomarkers values in CTRCD “+” group are colored in pink, and in light purple for CTRCD “−“ group.)

Exploring the chosen conventional biomarker panel performance, we aimed to select biomarkers with satisfactory ability for diagnosing asymptomatic CTRCD. We analyzed the biomarkers’ dynamics through CTRCD groups (with developed cardiotoxicity (CTRCD“+”, *n* 34; and without, CTRCD“−”, *n* 86) at specific time points (visits once in three months). As stated previously, we put into operation six biomarkers—cTnI, BNP, CRP, MPO, Gal-3, and D-dimer. The affordability considerations prevailed when planning the panel, as three of these biomarkers were included in the in-country BC protocol [34]. The graphical presentation of these biomarkers’ dynamics within 12 months of monitoring is displayed in Figure 2.

We used a Gaussian family-based generalized linear model (GLM) where the *x*-axis reflects visits, i.e., month_3 means visit 2, month_6 means visit 3, month_9—visit 4, and month_12—visit 5. The *y*-axis displays Log2-transformed biomarkers values. The GLM results provided valuable information about the relationships between biomarker levels and time points (visits) and the influence of different cardiotoxicity groups, “+” and “−”.

#### 3.3.1. Key Findings on BNP Levels across the CTRCD Groups (Graph A)

Baseline BNP levels: the model estimated the baseline BNP level (intercept) to be 5.64252 (*p* < 0.001), indicating that there was a substantial difference in BNP levels at the outset.

Temporal effects: at visit 2 (month_3), the estimated impact on BNP levels was 0.03006, but this change was not statistically significant (*p* = 0.734). However, BNP levels exhibited significant increases at visit 3 (month_6) with an estimated effect of 0.30506 (*p* = 0.001), visit 4 (month_9) with an impact of 0.34431 (*p* = 0.0001), and visit 5 (month_12) with a notable effect of 0.55724 (*p* < 0.001).

CTRCD group effects: the coefficient for CTRCD“−” was −0.05156, indicating a slight decrease in BNP levels in this group, but this effect was not statistically significant (*p* = 0.661).

Statistical analysis suggested that BNP levels changed significantly, particularly at visits 2–5, with a trend to increase mainly in the group CTRCD“+”. The dispersion parameter for the Gaussian family was estimated to be 0.3355248. The model’s fit assessment showed a notable reduction in residual deviance (197.96) compared to the null deviance (237.02), indicating that the model fits the data well.

#### 3.3.2. Key Findings on CRP Levels across the CTRCD Groups (Graph B)

Baseline CRP levels: the model estimated the baseline CRP level (intercept) to be 2.370805 (*p* < 0.001), signifying a substantial difference in CRP levels at the start of the study.

Temporal effects: at visit 2 (month_3), the estimated impact on CRP levels was 0.382840 (*p* = 0.095). Similarly, for visit 3 (month_6) and visit 5 (month_12), the estimated effects of 0.183424 and 0.017104, respectively, were not statistically significant (*p* > 0.05). Visit 4 (month_9) exhibited a negligible impact of −0.044046, which was also insignificant (*p* = 0.848).

CTRCD group effects: the coefficient for CTRCD“−” group was 0.254687, indicating a slight increase in CRP levels in this group, but this effect was not statistically significant (*p* = 0.403).

Interaction effects: none of the interaction terms was statistically significant (*p* > 0.05), suggesting that the influence of CTRCD groups did not differ significantly over the study period.

Overall, CRP levels did not exhibit substantial changes over time or significant associations with CTRCD groups during the study period. The dispersion parameter for the Gaussian family was estimated to be 2.254238. The model fit assessment showed a slight reduction in residual deviance (1330.0) compared to the null deviance (1363.1), indicating that the model reasonably fit the data.

#### 3.3.3. Key Findings on D-Dimer Levels across the CTRCD Groups (Graph C)

Baseline D-dimer levels: the model estimated the baseline D-dimer level (intercept) to be 0.1179 (*p* = 0.404). This suggests that there were no substantial differences in the biomarker levels at the beginning of the study.

Temporal effects: at visit 2 (month_3), there was a notable decrease in D-dimer levels within the group CTRCD“–”, with an estimated effect of −0.3906, approaching statistical significance (*p* = 0.051). At visits 3–5, fluctuation was observed, with an estimated effect of −0.5288 for visit 3 (*p* = 0.008), −0.3903 (*p* = 0.051) for visit 4, and with an effect of −0.7189 (*p* < 0.001) at visit 5. These findings suggest unstable trends in D-dimer levels over the study period.

CTRCD group effects: the coefficient for CTRCD“−” was −0.3901, indicating a negative impact on D-dimer levels, but this effect was not statistically significant (*p* = 0.142).

Interaction effects: month_6:CTRCD“+” had a statistically significant positive impact of 0.8743 at visit 3 (*p* = 0.020). However, the other interaction terms were not statistically significant (*p* > 0.05).

D-dimer levels exhibited a fluctuating trend over the study period. While there was no significant difference in D-dimer levels associated with CTRCD groups, a specific effect at visit 3 was observed in those with developed cardiotoxicity six months after the treatment onset. The dispersion parameter for the Gaussian family was estimated to be 1.711856, and the model exhibited a reasonable fit to the data, as indicated by the reduction in residual deviance (1010.0) compared to the null deviance (1047.1).

#### 3.3.4. Key Findings on Gal-3 Levels across the CTRCD Groups (Graph D)

Baseline Gal-3 levels: the model estimated the baseline Gal-3 level (intercept) to be 3.9246 (*p* < 0.001). This suggests the presence of measurable Gal-3 levels at the beginning of the study.

Temporal trends: noteworthy temporal trends in Gal-3 levels were observed. At visit 2 (month_3), there was a significant increase in Gal-3 levels, with an estimated effect of 0.2133 (*p* = 0.021). Furthermore, Gal-3 levels continued to rise at visit 3 (month_6) and visit 4 (month_9), with estimated effects of 0.3914 (*p* < 0.001) and 0.5419 (*p* < 0.001), respectively. By visit 5 (month_12), the Gal-3 levels remained elevated, though to a lesser extent, with an estimated effect of 0.3682 (*p* < 0.001).

Effect of CTRCD groups: the coefficient for CTRCD“−” was 0.0739, indicating a positive effect on Gal-3 levels, but this effect was not statistically significant (*p* = 0.546). It suggests that the CTRCD“−” group did not significantly affect Gal-3 levels.

Interaction effects: none of the interaction terms were statistically significant (*p* > 0.05). This suggests that the effect of CTRCD groups on Gal-3 levels remained consistent across different time points.

The statistical analysis highlighted a substantial increase in Gal-3 levels over the first nine months of the study, with a gradual decrease by month 12. The influence of CTRCD groups on Gal-3 levels was not statistically significant, and this effect remained constant over time. The dispersion parameter for the Gaussian family was estimated to be 0.3651235, and the model displayed a strong fit for data, with a reduction in residual deviance (215.42) compared to the null deviance (232.65).

#### 3.3.5. Key Findings on cTnI Levels across the CTRCD Groups (Graph E)

Intercept: the intercept, representing the baseline cardiac troponin level, was estimated at −3.06705 (*p* < 0.001), indicating a significant baseline difference between the biomarker values in CTRCD groups.

CTRCD group effects: the coefficient for the CTRCD“+” group was estimated at 0.12844 (*p* = 0.351), suggesting that this group had slightly higher troponin levels, though insignificant compared to the group CTRCD“−”.

Interaction effects: while some interactions, such as month_3:CTRCD“−” and month_6:CTRCD“−” showed potential trends, they were not statistically significant (*p* > 0.05).

Our analysis indicated a baseline difference in cardiac troponin levels, with some variations over time and among CTRCD groups. However, these differences were not always statistically significant. The dispersion parameter for the Gaussian family was estimated to be 0.4618251. The goodness of fit was assessed by comparing null deviance (278.19) and residual deviance (272.48) with an associated Akaike’s information criterion (AIC) value of 1251.1.

#### 3.3.6. Key Findings on MPO Levels across the CTRCD Groups (Graph F)

Baseline MPO levels: the model estimated the baseline MPO level (intercept) to be 0.2854 (*p* < 0.001). This suggests that at the start of the study, there were measurable levels of MPO.

Temporal effects: at visit 3 (month_6), there was a significant decrease in MPO levels, with an estimated effect of −0.0448 (*p* = 0.018). In contrast, the other time points, visit 2 (month_3), visit 4 (month_9), and visit 5 (month_12), did not exhibit significant changes in MPO levels.

Interaction effects: none of the interaction terms were statistically significant (*p* > 0.05). This suggests that the effect of CTRCD groups on MPO levels remained consistent across different time points.

The dispersion parameter for the Gaussian family was estimated to be 0.0154242, and the model demonstrated a satisfactory fit to the data, as indicated by the reduction in residual deviance (9.1003) compared to the null deviance (9.2554).

Among the six biomarkers explored, only BNP showed a reliable pattern of steady increase within a year of the treatment starting. The BNP median values have been compared between the CTRCD groups from visit to visit; data are presented in Table 3. By visit 4, i.e., nine months after the treatment commencement, statistically significant differences between CTRCD groups on BNP levels were observed.

### 3.4. Designing a Predictive Model for CTRCD Risk Evaluation during or by the End of Treatment before Follow-Up

Clinical risk factors play a crucial role in the baseline CV risk stratification, which, provided correctly performed, can significantly prevent unfavorable outcomes of chemotherapy in BC patients [17]. We performed univariate and multivariate logistic regression analyses to disclose the factors significantly associated with eventual cardiotoxicity (Table 4) for further building a nomogram.

**Figure 3:**
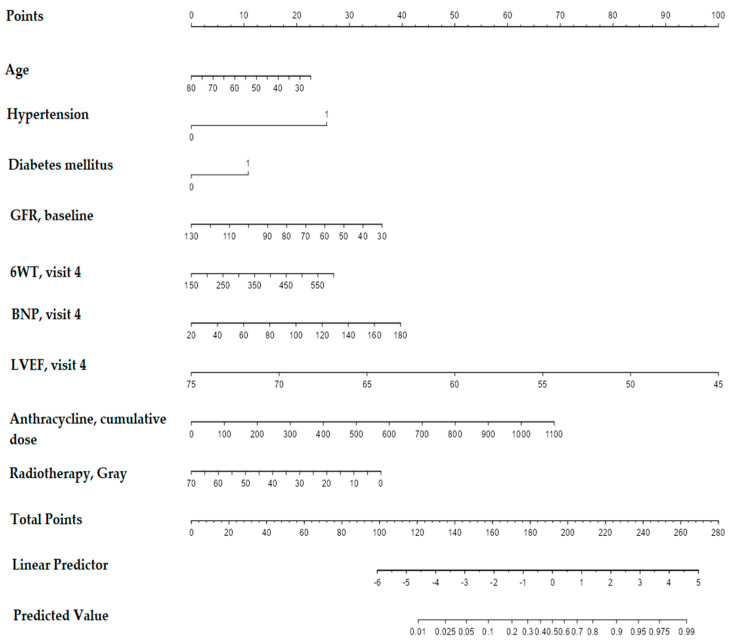
Nomogram for predictive model.

To build the nomogram, seventy percent of the 120 patients were randomly selected as the training cohort to develop the prediction models, and the remaining 30% were allocated to an internal validation cohort. Univariate logistic regression analysis unveiled variables significantly correlated (*p* < 0.05) with CTRCD in the training group: age, BMI, GFR levels, baseline CV risk, baseline 6WT, arterial hypertension, diabetes mellitus, anthracycline cumulative doses, etc. In addition, some clinically essential valuables approached statistical significance, such as intake of cardiac protectors, doses of irradiation received, and baseline LVEF. These variables were then entered into a multivariate logistic regression model. It is advised that variable selection should focus more on clinical knowledge than statistical selection methods alone [43]. The model was adjusted for all predictors of high clinical significance. In our multivariate analysis (Table 4), only two variables, anthracycline cumulative doses (OR 1.002 [1.000; 1.004]) and arterial hypertension (OR 5.178 [2.042; 13.131]), reached statistical significance, and therefore we utilized significant variables obtained in univariate analysis. Thus, for establishing the model, we operated with the following predictors: age, arterial hypertension, diabetes mellitus, baseline GFR, 6WT measured at visit 4, BNP values measured at visit 4, LVEF levels at visit 4, i.e., nine months after the treatment onset, total dose of irradiation received, and anthracycline cumulative doses. We added BNP values as, in our study, BNP was the only biomarker that showed satisfactory performance regarding asymptomatic CTRCD. As we designed the model for use before the upcoming follow-up and in settings where speckle tracking echocardiography is unavailable, we introduced such predictors as 6WT and LVEF measured after visit 4, i.e., closer to the follow-up, when the issues of further patient management arise. We removed correlated variables, which did not affect the model’s performance, as they measured the same underlying information as the variable to which they correlated [43]. For instance, we removed variables such as systolic BP and glucose values as they related to hypertension and diabetes mellitus. The nomogram (Figure 3) was built to present the model for evaluating the patient’s situation regarding CTRCD risk during or at the end of treatment before follow-up. The results were expressed as percentages (%).

The final score, which predicts the risk of developing CTRCD, is calculated by adding the scores of each variable. For example, a 70-year-old patient (4 points) with arterial hypertension (26 points) and diabetes mellitus (11 points), with a GFR of 82 mL/min/1.73 m^2^ (17.5 points), who performed a 368 m 6WT (13 points) at visit 4, with a BNP value at visit 4 of 93 pg/mL (19 points), an LVEF level at visit 4 of 57% (57.5 points), and a cumulative dose of doxorubicin 675 mg/kg (42 points), with a total dose of preceding radiation treatment 46 Gy (12.5 points), scored in sum 202.5 points. This score evidences the predicted CTRCD risk of 70%.

Another example taken from the cohort without signs of CTRCD (*n* 86): patient 47 years old (15 points), without arterial hypertension (0 points) and diabetes mellitus (0 points), with GFR of 109 mL/min/1.73 m^2^ (7.5 points), who performed a 365 m 6WT at visit 4 (14 points), with BNP level at visit 4 of 43.15 pg/mL (5 points) and LVEF level of 57% (57.5 points), with anthracycline cumulative dose of 400 mg/kg (25 points), with preceded radiotherapy of 46 Gy (12.5 points), scored summarily 136.5 points. The probable risk of CTRCD—3%. The risk was predicted correctly, as this patient did not develop CTRCD within 12 months of monitoring.

**Figure 4:**
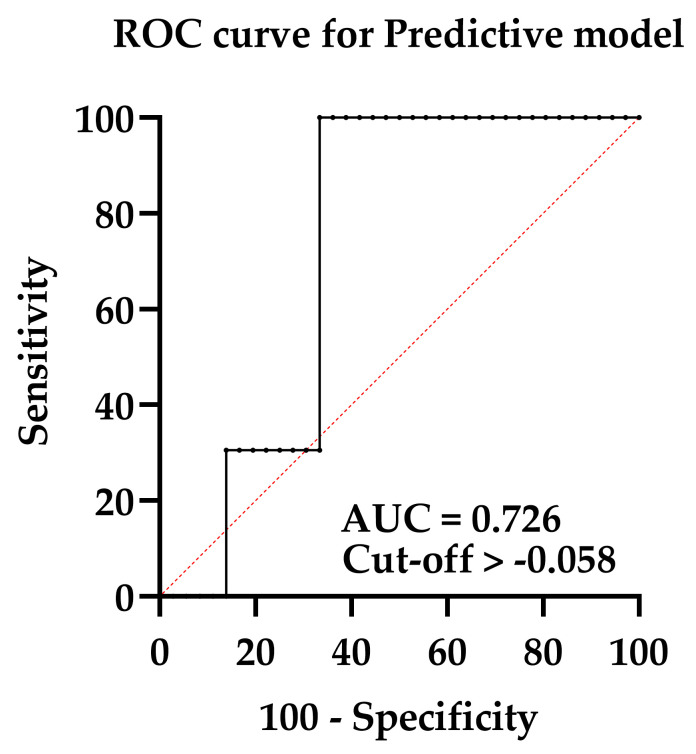
ROC curve for assessing the predictive model.

The correspondence of the training and validation cohorts, i.e., the predictive efficacy of the model, was assessed by three methods: construction of the ROC curve, Hosmer–Lemeshow statistics, and building of the calibration plot. The area under the ROC curve describes the ability of the model to distinguish between those patients who have the outcome of CTRCD“+” and those who do not, which was assessed by calculating the model’s sensitivity and 1-specificity. The ROC curve for the presented nomogram is displayed in Figure 4.

**Figure 5:**
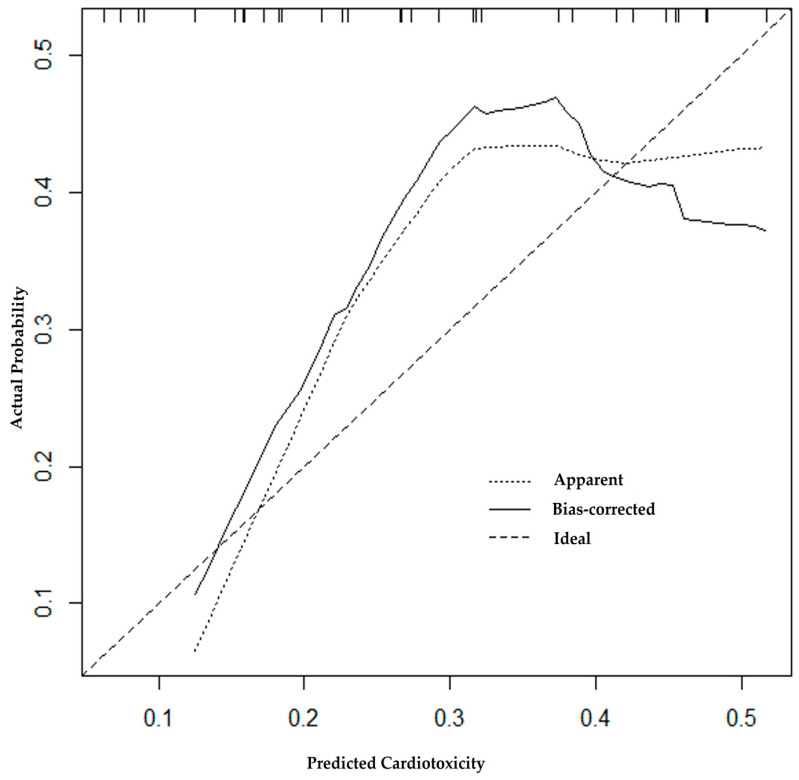
Calibration plot for assessing the predictive model.

The AUC was 0.72 (95% CI 0.59; 0.86), evidencing the satisfactory predictive ability of the model. The model’s sensitivity was 100% (95% CI 90.36; 100.0) at a specificity of 66.67% (95% CI 50.33; 79.79). The model’s PPV appeared to be 54.1% [95% CI 47.13; 60.91]; PVN 100% [95% CI 94.64; 100.00]. The calibration plot for the model is presented in Figure 5.

The Hosmer–Lemeshow goodness-of-fit test was performed to evaluate the model calibration. The Chi-Sqr. statistic was 2.0544; df 8; *p*-value 0.9793. The calibration plot describes the consistency between the model-predicted probabilities and the observed probabilities of CTRCD“+”, evaluated by the Hosmer–Lemeshow test. The plot showed satisfactory agreement between predicted and observed (actual) chances (*p* = 0.98).

## 4. Discussion

Exploring cardiotoxicity issues gained a great scope following improved therapeutic options in breast cancer patients. Meanwhile, research studying this problem, particularly the issues of cardiotoxicity mitigation or its management in breast cancer survivors, is still limited [44]. As the incidence of BC ranks first globally, a community of women diagnosed with breast cancer comprises a substantial proportion of cancer survivors.

Reportedly, CTRCD occurs in approximately 10% of patients [45], but the incidence varies depending on many factors. According to South Korean retrospective research with *n* 1200, 134 out of 439 BC patients with LVEF data have been classified into CTRCD, which means that CTRCD affected a quarter of all BC patients or even more [46]. For anthracycline-based therapy, the risk of cardiovascular complications increases as the cumulative dose administered increases. However, there is a different level of risk for each patient scheduled for anthracycline therapy: patients older than 65 years with prior or concurrent chest irradiation, pre-existing heart disease, or already known cardiovascular risk factors have an increased risk for cardiotoxicity [47]. In the Spanish research, 27.4% of patients were asymptomatic in the cohort of 113 individuals, where breast cancer and anthracyclines were the most frequent cancer and therapy [48]. Our mixed cohort (*n* 120, where 98 received anthracycline therapy) detected 34 cases of asymptomatic cardiotoxicity (28.3%).

Preconditions underlying CTRCD development are essential to explain the growing incidence of CTRCD. Many researchers highlight the role of clinical factors, particularly pre-existing CV diseases. The role and influence of pre-existing CV diseases on the risk of cardiotoxicity during antineoplastic treatment were stated in the latest ESC Cardio-Oncology Guidelines 2022 [17]. As shown in the mentioned retrospective research [46], 45.7% of patients had pre-existing cardiovascular diseases, and 91.6% were classified as very high risk for cardiotoxicity in the pre-existing CVDs group. Kobat et al. [49] showed that cardiotoxicity risk was increased in non-small-cell lung cancer patients with prior diabetes mellitus (OR = 1.93, *p* = 0.038), and the presence of baseline cardiovascular disease (OR = 2.03, *p* = 0.018). In our cohort, patients who developed CTRCD differed from those without CTRCD by the presence of previously existing diseases: hypertension (*p* < 0.001), diabetes mellitus (*p* 0.001), and ischemic heart disease (0.023). According to Kazakhstan researchers, BC mortality was associated with women diagnosed with diabetes (HR 1.2) and arterial hypertension (HR 0.4) [3].

As known, asymptomatic cardiotoxicity can only be diagnosed via imaging methods, and systematic monitoring is required. Of the two methods of LVD visualization (TTE and speckle tracking), GLS monitoring has been recognized as the best method for detecting asymptomatic cardiotoxicity. One of the main limitations of LVEF is the significant coefficient of variation, which limits its sensitivity for detecting small changes in LV function. In contrast, declines in GLS can occur without substantial changes in LVEF [50]. In the pilot study on the performance of imaging techniques to detect subclinical cardiotoxicity, two-dimensional speckle-tracking imaging appeared to be more advantageous than LVEF measured by three-dimensional echocardiography. In addition, some indices, such as TAPSE (tricuspid annular plane systolic excursion), are reported to be more sensitive for asymptomatic CTRCD detection [51]. However, when searching for a correlation of STE (GLS) with traditional biomarkers in predicting cardiotoxicity among pediatric patients, the ROC analysis did not find GLS to be a significant independent predictor of cardiotoxicity (*p* > 0.05) [52]. Nevertheless, unlike STE, three-dimensional TTE is relatively cost-effective and available and, as such, remains in the range of tools for CTRCD diagnosing. The study mentioned above showed that in HER2+ early BC, not highly sensitive primary biomarkers but LVEF trajectory allowed for a dynamic assessment of cardiotoxicity risk [32]. In our study, LVEF measured by TTE failed to show a >10% decline from baseline to <53% for 12 months of monitoring, evidencing that TTE alone cannot be a reliable method except in combination with other tools and approaches.

We examined trends in six biomarker performances across CTRCD“+” and “−” groups to select an acceptable indicator of incrementally increasing cardiotoxicity (cTnI, BNP, CRP, MPO, Gal-3, and D-dimer). Only BNP showed a consistent and statistically significant upward trend from visit to visit, particularly in the CTRCD“+” sample. This trend was observed irrespective of the treatment type, though BNP evidenced the highest performance within the anthracycline treatment group in our study. BNPs elevation, irrespective of BNP type, was widely recognized to be associated with cardiotoxicity during anthracycline chemotherapy and may even be more sensitive for predicting cardiotoxicity than echocardiography [53,54].

Contrary to expectations, cTnI in our study was not a reliable marker despite numerous papers evidencing the high efficacy of troponins in CTRCD. As known, 2020 ESMO consensus recommendations defined subclinical cardiac dysfunction as an absolute decrease from baseline in the GLS of −5% or a relative decrease from baseline in the GLS of −12% or troponins elevation from baseline, highlighting the leading role of troponins [55]. Highly sensitive types of troponins are characterized by higher precision and earlier rise. In particular, Hs-cTnI concentrations are more sensitive to coronary artery disease and ischemic outcomes [56]. Japanese researchers showed that early relative worsening of >15% in GLS was significantly associated with hsTnI elevation [57]. As shown recently, hs-cTnT testing can improve the accuracy of CTRCD clinical diagnosis [58].

In addition to cTnI, MPO, CRP, D-dimer, and Gal-3 also failed to manifest as reliable predictors of asymptomatic CTRCD in our study, contradicting the reports of other researchers. In studies, MPO was strongly associated with an increased risk of coronary artery disease and acute HF, being a promising biomarker of cancer therapy-related cardiotoxicity in BC patients treated with anthracyclines and trastuzumab [20,59,60,61]. Reportedly, high D-dimer level at baseline was an independent predictor of the development of CTRCD [62]. Our statistical analysis highlighted a substantial increase in Gal-3 levels over the first nine months of the study, with a gradual decrease by month 12, i.e., the Gal-3 trajectory was not steadily rising. Although Gal-3 holds promise for predicting cardiotoxicity, we agree with other authors that the monitoring value of galectin-3 requires further study [60].

We have attempted to design a predictive model based on essential data from patient monitoring that can be applied to assessing the patient’s chance of developing cardiotoxicity during the treatment, particularly before the upcoming follow-up. The model should contribute to the timely modification of chemotherapy to prevent unfavorable outcomes. The model includes imaging, laboratory, and observational clinical data at different stages, as CTRCD risk is a dynamic variable, and the risk changes throughout the care pathway [17]. Presumably, this model can have good prospects to be utilized in settings where the cardio-oncology service has not been established yet and lacks STE monitoring. Along with that, there are some caveats to using the model. Our model provides risk estimations based on the available data, but these estimations are not always guaranteed to be correct due to the limited predictive accuracy (PPV = 54.1%; PVN 100%). Though the model has demonstrated relatively satisfactory discrimination (AUC = 0.726) and passed internal validation (GOF *p* = 0.98), it has not undergone clinical validation yet. One should consider the model’s predictions as one of the sources of information that should be used in conjunction with other established clinical assessments.

We revealed inverse proportionality between the age and scores in the model. Older patients can receive lower scores than those at younger ages. We suppose this is related to the following circumstances: the model was primarily designed for those who received anthracycline therapy, and younger age at diagnosis is a significant risk factor, at least for pediatric patients on anthracycline therapy [63]. Chinese researchers showed that the risk of CVD mortality was increased among breast cancer patients at the age of follow-up 30–64 years and was mainly activated by heart diseases [7]. In addition, predictors are unequal by weight and significance within the hierarchy. For instance, hypertension and anthracycline cumulative dose are the most weighty predictors, and age does not play a leading role when such predictors arise in the patient’s profile.

Some features of this study can be referred to as positive aspects. To our knowledge, we are the first researchers who tried to develop a predictive model that can serve as a practical alternative when advanced echocardiographic methods are unavailable. Despite the limited predictive accuracy, the model has passed internal validation and can be applied with other clinical tools. Second, we provided a direct comparison of six conventional biomarkers used in everyday practice and confirmed the leading role of NPs in subclinical CTRCD diagnosis. We also figured out and demonstrated the need to utilize highly sensitive troponins for early diagnosis of cardiotoxicity.

The study had inevitable limitations. First was the substantial heterogeneity of our cohort, where 98 patients received anthracyclines, 12—trastuzumab, and 10—combined treatments. Such inequity significantly limited the chance of in-depth analysis across treatment groups, potentially leading to bias. In addition, we presented data without follow-up, whereas the weight of clinical risk factors can change over time.

## 5. Conclusions

We detected 28.3% of patients with asymptomatic (subclinical) CTRCD in the cohort with *n* 120, and many patients were predisposed to develop CTRCD due to pre-existing CV diseases. The leading role of NPs in subclinical CTRCD diagnosis was confirmed in the research. The predictive model based on essential data from patient monitoring can be applied to assessing the patient’s chance of developing cardiotoxicity in settings lacking speckle tracking, the gold standard of current echocardiography. The presented model requires further research and clinical validation.

## Figures and Tables

**Table 1:** The baseline characteristics of patients with and without developed subclinical cardiotoxicity.

Variables	Total, *n* = 120	Cardiotoxicity (−) *n* = 86	Cardiotoxicity (+) *n* = 34	*p*-Value
Age, years	55.5 ± 11.1	52.5 ± 11.5	57.9 ± 8.9	*0.018*
BMI*, kg/m^2^	27.3 (23.2; 30.9)	26.1 (23.0; 30.9)	28.6 (25.6; 31.3)	0.056
SBP*, mmHg	120.0 ± 17.9	117.0 ± 17.0	127.3 ± 18.0	*0.002*
Charlson comorbidity index, scores	5 (3; 6)	5 (3; 6)	5 (4; 6)	*0.047*
Glucose, mmol/L	5.2 (4.7; 5.6)	5.2 (4.7; 5.5)	5.5 (4.9; 6.0)	0.018
Cholesterol, mmol/L	5.3 ± 0.85	5.3 ± 0.86	5.1 ± 0.84	0.297
Creatinine, µmol/L	67.6 (59.2; 77.2)	67.4 (58.8;76.5)	67.7 (62.0;78.4)	0.467
Egfr*, mL/min/1.73 m^2^	90.7 ± 17.6	92.7 ± 17.8	85.6 ± 16.1	0.070
Baseline 6WT	423.8 ± 60.5	431.8 ± 59.1	403.5 ± 59.9	*0.038*
Baseline LVEF, %	59.8 ± 3.6	60.1 ± 3.7	58.8 ± 3.3	0.074
Baseline GLS, %	−18.2 (−16.8; −19.5)	−18.2 −16.9; −19.5)	−17.9 (−16.5; −19.4)	0.835
Baseline cTnI, ng/mL	0.10 (0.10;0.10)	0.10 (0.10; 0.10)	0.10 (0.10; 0.22)	0.337
Baseline BNP, pg/mL	48.9 (35.5; 65.1)	49.3 (35.8; 65.5)	48.9 (35.3; 60.5)	0.628
Baseline CRP, mg/L	5.1 (2.8; 11.5)	4.8 (2.7; 10.3)	5.3 (3.3; 12.1)	0.389
Baseline D dimer, mg/L	0.90 (0.53; 2.08)	1.02 (0.54; 2.25)	0.73 (0.52; 1.49)	0.133
Baseline MPO, pmol/L	120 (100; 301)	120 (100; 164)	120 (120; 301)	0.560
Baseline Gal-3, ng/L	14.7 (11.4; 21.1)	14.7 (11.4; 20.5)	14.7 (11.4; 23.3)	0.596
Anthracycline, cumulative doses	408.9 ± 228.1	382.3 ± 233.4	476.0 ± 201.8	0.045
Radiotherapy, GY*	33.9 ± 15.9	34.5 ± 16.1	32.4 ± 15.6	0.392
Baseline CV* risk, *n* (%)				0.011
High	7 (5.83)	3 (3.5)	4 (11.8)	
Moderate	46 (38.33)	28 (32.5)	18 (52.9)	
Low	67 (55.83)	55 (64.0)	12 (35.3)	
Heredity, *n* (%)	14 (11.7)	11 (12.8)	3 (8.8)	0.542
Menopause, *n* (%)	79 (65.8)	53 (61.6)	26 (76.5)	0.122
Localization:				0.174
Right, *n* (%)	52 (43.3)	35 (50.0)	17 (50.0)	
Left	65 (54.2)	51 (50.0)	14 (41.2)	
Both sides	3 (2.5)	-	3 (8.8)	
Clinical stage, *n* (%):				0.971
I	6 (5.0)	5 (5.8)	1 (2.9)	
IIA	44 (36.7)	32 (37.2)	12 (35.3)	
IIB	54 (45.0)	37 (43.0)	17 (50.0)	
IIIA	5 (4.2)	4 (4.7)	1 (2.9)	
IIIB	11 (9.1)	8 (9.3)	3 (8.8)	
Tumor histotype, *n* (%):				0.522
1-Invasive carcinoma unspecified	84 (70.0)	57 (66.3)	27 (79.4)	
2-Invasive ductal carcinoma	31 (25.8)	25 (29.1)	6 (17.7)	
3-Invasive lobular cancer	4 (3.3)	3 (3.5)	1 (2.9)	
4-Angiosarcoma	1 (0.83)	1 (1.1)	-	
Breast cancer types, *n* (%):				0.442
Nodular cancer	101 (84.2)	72 (83.7)	29 (85.4)	
Diffuse forms	19 (15.8)	14 (16.3)	5 (14.6)	
Immunohistochemistry, *n* (%):				0.065
TNBC*	19 (15.8)	17 (19.8)	2 (5.9)	
Luminal A type	33 (27.5)	23 (26.7)	10 (29.4)	
Luminal B (positive)	17 (14.2)	12 (14.0)	5 (14.7)	
Luminal B (negative)	43 (35.8)	26 (30.2)	17 (50.0)	
HER2-positive	8 (6.7)	8 (9.3)	-	
Smoking, *n* (%)	16 (13.3)	11 (12.8)	5 (14.7)	0.781
Hypertension, *n* (%)	61 (50.8)	35 (40.7)	26 (76.5)	*<0.001*
Diabetes mellitus, *n* (%)	16 (13.3)	6 (7.0)	10 (29.4)	*0.001*
Ischemic heart disease, *n* (%)	2 (1.7)	-	2 (5.9)	*0.023*
Cardioprotective medications, if prescribed, *n* (%)	61 (50.8)	39 (45.3)	22 (64.7)	0.056
Treatment administered, *n* (%):				0.269
Anthracyclines	98 (81.7)	68 (79.1)	30 (88.2)	
Combined	10 (8.3)	7 (8.1)	3 (8.8)	
Trastuzumab	12 (10)	11 (12.8)	1 (2.9)	

BMI*—body mass index; SBP*—systolic blood pressure; Egfr*—glomerular filtration rate; GY*—Gray, i.e., radiotherapy total doses measured in Gray; CV*—cardiovascular; TNBC*—triple-negative breast cancer.

**Table 2:** Left ventricular ejection fraction (LVEF)/global longitudinal strain (GLS) data for 12 months of monitoring across CTRCD groups.

Visits	CTRCD(+), M (IQR), *n* = 34	CTRCD(−), M (IQR), *n* = 86	*p*-Value
GLS (%):			
1	−18.1 (−19.4; −16.5)	−18.2 (−19.5; −16.9)	0.835
2	−16.6 (−17.6; −15.5)	−17.75 (−18.7; −16.7)	0.003
3	−15.7 (−16.7; −14.1)	−17.6 (−18.8; −16.4)	<0.000
4	−14.9 (−16.1; −13.2)	−17.4 (−18.4; −16.4)	<0.000
5	−13.4 (−14.7; −12.2)	−17.1 (−18.4; −15.7)	<0.000
ANOVA Chi Sqr. (*n* = 34, df = 4) 88.846; *p* < 0.00001; Coeff. of concordance 0.653; aver. Rank r = 0.643.		ANOVA Chi Sqr. (*n* = 86, df = 4) 16.945; *p* 0.002; Coeff. of concordance 0.049; aver. Rank r = 0.038.	
LVEF (%):			
1	58.0 (57.0; 61.0)	60.0 (58.0; 62.0)	0.075
2	56.0 (55.0; 59.0)	58.0 (56.0; 60.0)	0.004
3	56.0 (55.0; 57.0)	58.0 (56.0; 60.0)	0.0003
4	56.0 (55.0; 58.0)	58.0 (56.0; 59.0)	0.0001
5	56.0 (54.0; 57.0)	57.0 (56.0; 59.0)	0.0003
ANOVA Chi Sqr. (*n* = 34. df = 4) 35.98; *p* < 0.00000; Coeff. of concordance = 0.264; aver. Rank r = 0.242.		ANOVA Chi Sqr. (*n* = 86. df = 4) 39.92; *p* < 0.00000; Coeff. of concordance = 0.116; aver. Rank r = 0.106.	

**Table 3:** BNP median values within 12 months of monitoring across CTRCD groups.

Visits	CTRCD(+), M (IQR), *n* = 34	CTRCD(−), M (IQR), *n* = 86	*p*-Value
1	48.9 (35.3; 60.5)	49.3 (35.7; 65.5)	0.629
2	56.8 (42.7; 68.3)	49.0 (37.2; 64.1)	0.104
3	65.3 (48.0; 89.7)	61.7 (47.9; 83.1)	0.463
4	78.6 (62.5; 97.9)	64.4 (52.0; 81.5)	0.006
5	87.8 (71.8; 114.5)	67.9 (58.9; 89.00)	0.001
ANOVA Chi Sqr. (*n* = 34; df = 4) 38.353; *p* < 0.00000; Coeff. of concordance = 0.282; aver. Rank r = 0.260.		ANOVA Chi Sqr. (*n* = 86; df =4) 61.455; *p*< 0.00000; Coeff. of concordance = 0.179; aver. Rank r = 0.169.	

**Table 4:** Factors contributing to the development of eventual cardiotoxicity.

Variables, *n* 120	Univariate Analysis		Multivariate Analysis	
	OR (95% CI)	*p*-Value	aOR (95% CI)	*p*-Value
Age, years	1.049 (1.008;1.092)	*0.019*		
Body mass index, kg/m^2^	1.059 (0.998;1.124)	*0.051*		
SBP, mmHg	1.032 (1.009;1.056)	*0.006*		
Charlson comorbidity index, scores	1.166 (0.960;1.416)	0.121		
Cholesterol, mmol/L	0.798 (0.492;1.295)	0.361		
Glucosae, mmol/L	1.479 (1.030;2.124)	*0.034*		
Creatinine, µmol/L	1.018 (0.988;1.049)	0.232		
eGFR, mL/min/1.73 m^2^	0.977 (0.954;1.00)	*0.048*		
Baseline 6WT, meters	0.992 (0.985;0.999)	*0.024*		
Baseline cTnI, ng/mL	7.674 (0.065;903.591)	0.402		
Baseline BNP, pg/mL	1.011 (0.997;1.025)	0.116		
Baseline CRP, mg/L	1.002 (0.983;1.022)	0.822		
Baseline D-dimer, mg/L	0.781 (0.568;1.074)	0.129		
Baseline MPO, U/mL	0.997 (0.986;1.009)	0.650		
Baseline Gal-3, ng/mL	1.023 (0.975;1.075)	0.351		
Baseline LVEF,%	0.901 (0.801;1.013)	*0.080*		
Baseline GLS,%	1.037 (0.880;1.222)	0.663		
Anthracycline, cumulative dose	1.002 (1.000;1.004)	*0.046*	1.002 (1.000; 1.004)	*0.030*
Baseline cardiotoxicity risk:				
Low risk	Reference			
Moderate	2.569 (1.094;6.031)	*0.030*		
High	5.641 (1.123;28.345)	*0.036*		
Heredity	0.660 (0.172;2.529)	0.544		
Menopause	2.024 (0.820;4.996)	0.126		
Left side of the breast	0.565 (0.247;1.293)	0.177		
Smoking	1.176 (0.376;3.678)	0.781		
Hypertension	4.736 (1.922;11.667)	*0.001*	5.178 (2.042; 13.131)	*0.001*
Diabetes mellitus	5.556 (1.831;16.860)	*0.002*		
Cardioprotective medications, if prescribed	2.21 (0.97;5.02)	*0.058*		
Radiotherapy, total dose in Gy	0.992 (0.968;1.016)	*0.059*		
Radiotherapy, if received	0.987 (0.287;3.390)	0.983		

## Data Availability

The data obtained in this study will be openly available at [http://www.isrctn.com/ISRCTN12628444 (accessed on 21 July 2022)], and [http://osf.io/nykmw/ (accessed on 10 June 2022)].

## Supplementary Materials

All Supplementary Materials relevant to the presented study protocol are placed in a publicly available repository: [http://osf.io/nykmw/] (accessed on 10 June 2022).

## Abbreviations

ACE (inhibitors)—angiotensin-converting enzyme inhibitors; ARBs—angiotensin II receptor blockers; AUC—area under the curve; BC—breast cancer; BMI—body mass index; BNP—B-type natriuretic peptide; CHF—chronic heart failure; CRP—C-reactive protein; cTnI—cardiac troponin I; CTRCD—cancer therapy-related cardiac dysfunction; CVDs—cardiovascular diseases; ESC—European Society of Cardiology; Gal-3—galectin-3; GFR—glomerular filtration rate; GLS—global longitudinal strain; GOF—goodness-of-fit (test); HER2—human epidermal growth factor receptor 2; HF—heart failure; ICIs—immune checkpoint inhibitors; IHC—immunohistochemistry; KIs—small molecule protein kinase inhibitors; LVD—left ventricular dysfunction; LVEF—left ventricular ejection fraction; MPO—myeloperoxidase; PPV—positive predictive value; PVN—negative predictive value; ROC—receiver operating characteristic; SBP—systolic blood pressure; Sn—sensitivity; Sp—specificity; sST2—soluble suppression of tumorigenicity 2; STE—speckle tracking echocardiography; TNBC—triple-negative breast cancer; TTE—transthoracic echocardiography; 6WT—6 min walk test.
